# Artificial intelligence-driven decision support for patients with acute respiratory failure: a scoping review

**DOI:** 10.1186/s40635-025-00791-3

**Published:** 2025-08-08

**Authors:** Preeti Gupta, Alex K. Pearce, Thaidan Pham, Michael Miller, Korey Brunetti, Karen Heskett, Atul Malhotra, Anoop Mayampurath, Majid Afshar

**Affiliations:** 1 Scripps Research, La Jolla, CA USA; 2 University of California San Diego, San Diego, CA USA; 3 University of Wisconsin-Madison, Madison, WI USA

## Abstract

**Background:**

Artificial intelligence (AI) has emerged as a promising tool for decision support in managing acute respiratory failure, yet its real-world clinical impact remains unclear. This scoping review identifies clinically validated AI-driven tools in this domain, focusing on the reporting of key evaluation quality measures that are a prerequisite for broader deployment.

**Eligibility criteria:**

Studies were included if they compared a clinical, human factors, or health systems-related outcome of an AI-driven intervention to a control group in adult patients with acute respiratory failure. Studies were excluded if they lacked a machine learning model, compared models trained on the same dataset, assessed only model performance, or evaluated models in simulated settings. A systematic literature search was conducted in PubMed, CINAHL, and EmBase, from inception until January 2025. Each abstract was independently screened by two reviewers. One reviewer extracted data and performed quality assessment, following the DECIDE-AI framework for early-stage clinical evaluation of AI-based decision support systems.

**Results:**

Of 5,987 citations, six studies met eligibility. The studies, conducted between 2012 and 2024 in Taiwan, Italy, and the U.S., included 40–2,536 patients. Four studies (67%) focused on predicting weaning from mechanical ventilation. Three (50%) of the studies demonstrated a statistically significant and clinically meaningful outcome. Studies met a median of 3.5 (IQR: 2.25–6.25) of the 17 DECIDE-AI criteria. None reported AI-related errors, malfunctions, or algorithmic fairness considerations. Only one study (17%) described user characteristics and adherence, while two (33%) assessed human–computer agreement and usability.

**Conclusions:**

Our review identified six studies evaluating AI-driven decision support tools for acute respiratory failure, with most focusing on predicting weaning from mechanical ventilation. However, methodological rigor for early clinical evaluation was inconsistent, with studies meeting few of the DECIDE-AI criteria. Notably, critical aspects such as error reporting, algorithmic fairness, and user adherence were largely unaddressed. Further high-quality assessments of reliability, usability, and real-world implementation are essential to realize the potential of these tools to transform patient care.

**Supplementary Information:**

The online version contains supplementary material available at 10.1186/s40635-025-00791-3.

## Introduction

Mortality due to acute respiratory failure (ARF) has been increasing by 3.4% annually, accounting for over 1.4 million deaths due to this condition between 2014 and 2018 in the United States (U.S.) [1]. Acute respiratory failure is both common and severe, contributing to more than 3 million hospitalizations in the U.S. in 2017, with over 1 million of those patients requiring mechanical ventilation [2]. Aside from an aging population, lack of sufficient resources, limited adherence to optimal ventilatory strategies, and heterogeneity of the condition contribute to poor outcomes [1–3].

Artificial intelligence (AI), which involves using computer systems to perform tasks that normally require human intelligence, has emerged as a promising tool to augment decision-making in medicine, with a growing number of clinical applications being developed [4]. In acute respiratory failure, these tools have the potential to increase workflow efficiency, promote guideline-directed care, and provide personalized recommendations tailored to distinct phenotypes [5–7]. For instance, an AI model trained to recommend optimal positive end-expiratory pressure, fraction of inspired oxygen, and ideal body weight–adjusted tidal volume using electronic health record data was associated with a lower estimated hospital mortality rate compared to observed outcomes under usual care, and increased time spent within optimal oxygen saturation and blood pressure targets [8]. Another model, developed to predict optimal allocation of intensive care unit nursing staffing, found that higher-than-predicted nurse staffing levels were associated with a lower risk of healthcare-associated infections in neonatal intensive care units [9].

However, despite encouraging simulations, most AI-driven prediction models do not transition to integrated real-world clinical decision support (CDS) systems. Reviews suggest fewer than one percent make the transition to tools that deliver clinical impact [10, 11]. The complexity of this task is highlighted by the "Five Rights" of clinical decision support: delivering the right information, to the right person, in the right format, through the right channel, and at the right time [12]. Thus, studies must not only develop the right information, i.e., an AI tool with high predictive accuracy, but also incorporate human factors and workflow integration to realize real-world benefit.

The primary objective of this review is to describe the landscape of AI-driven interventions for acute respiratory failure and assess the quality of their clinical evaluations. To achieve this, we systematically identified relevant studies and applied the Developmental and Exploratory Clinical Investigations of Decision support systems driven by Artificial Intelligence (DECIDE-AI) reporting guideline to each study, with an additional focus on the usability and implementation components of the framework [13]. Given the substantial variability in AI tool types, study designs, and clinical outcomes reported in the literature, a scoping review approach was selected as the most appropriate method to explore this evolving field, survey best practices, and inform future research priorities.

## Methods

### Eligibility criteria and protocol development

Studies were included if they met the following conditions: (1) incorporated an AI-driven clinical decision support (CDS) tool, defined as a tool that generates patient-specific predictions from a model trained using a machine learning algorithm; (2) tested an outcome in a clinical setting on adult patients with acute respiratory failure; (3) assessed a clinical, human factors or health system-related endpoint beyond predictive model performance; and (4) compared an outcome against a control group or usual care practice. Studies were excluded if the intervention was not based on a machine learning model—i.e. a model trained on data to identify patterns and make predictions, the comparison was a model trained on the same dataset as the intervention, the outcomes only tested model predictive performance, or the evaluation occurred in a simulated setting. Eligibility was also restricted to English language publications due to resource constraints. This study followed the Preferred Reporting Items for Systematic Reviews and Meta-Analyses (PRISMA) extension for scoping reviews and the completed checklist of required items is shown in Supplementary Table 1 [14]. This scoping review was not registered on PROSPERO due to ineligibility [15].

### Search strategy

The systematic literature search was conducted in PubMed, CINAHL, and Embase, from the inception of the database until January 7, 2025. Two health sciences librarians (K.B. and K.H.) collaborated with the research team to develop search strategies. Several MeSH headings and keyword terms for "Respiratory Insufficiency" were combined with the "Artificial Intelligence" MeSH term and several keyword terms for AI algorithms and related concepts (see Supplementary Table 2). Headings were not limited to major concepts to capture subheadings. No search limits were imposed on PubMed; for CINAHL and Embase searches, the academic journal limiter was used.

### Selection of citations and data extraction

Citations were loaded into Covidence^©^ software and were auto and manually de-duplicated [16]. Two pulmonary and critical care attending physicians (P.G. and A.K.P.) and two pulmonary and critical care fellows (T.P. and M.M.), all with experience or interest in artificial intelligence applications in medicine, reviewed the abstracts to determine eligibility for inclusion. Each abstract was independently evaluated by two reviewers, and inter-rater agreement was measured by proportion of agreement and Gwet’s AC1, which was selected for its reliability in measuring agreement when inclusion prevalence is low [17]. Conflicts were resolved either by consensus or third-person adjudication (M.A.). One reviewer conducted a full-text analysis of the selected studies, with concordance demonstrated by assigning a second reviewer to analyze > 20% of these studies. Data extraction and quality assessment were performed by one reviewer using a predefined extraction form (see Supplementary Table 3) and verified by a second reviewer. Extracted variables included study characteristics, settings, participant demographics, details of the AI-driven interventions, comparators, and outcomes. Descriptive statistics were performed using t-tests for continuous variables and Fisher’s exact test for categorical variables.

### Critical appraisal

Studies were assessed for quality according to the DECIDE-AI framework, specifically designed for early-stage clinical evaluation of artificial intelligence-based decision support systems [13]. The quantification of AI-specific reporting items addressed in each study facilitated the identification of patterns and themes related to readiness for clinical deployment across the reviewed literature. Each study was also screened to see if a performance bias assessment, such as Transparent Reporting of a multivariable prediction model for Individual Prognosis Or Diagnosis (TRIPOD), was conducted [18].

## Results

### Study selection

**Fig. 1:**
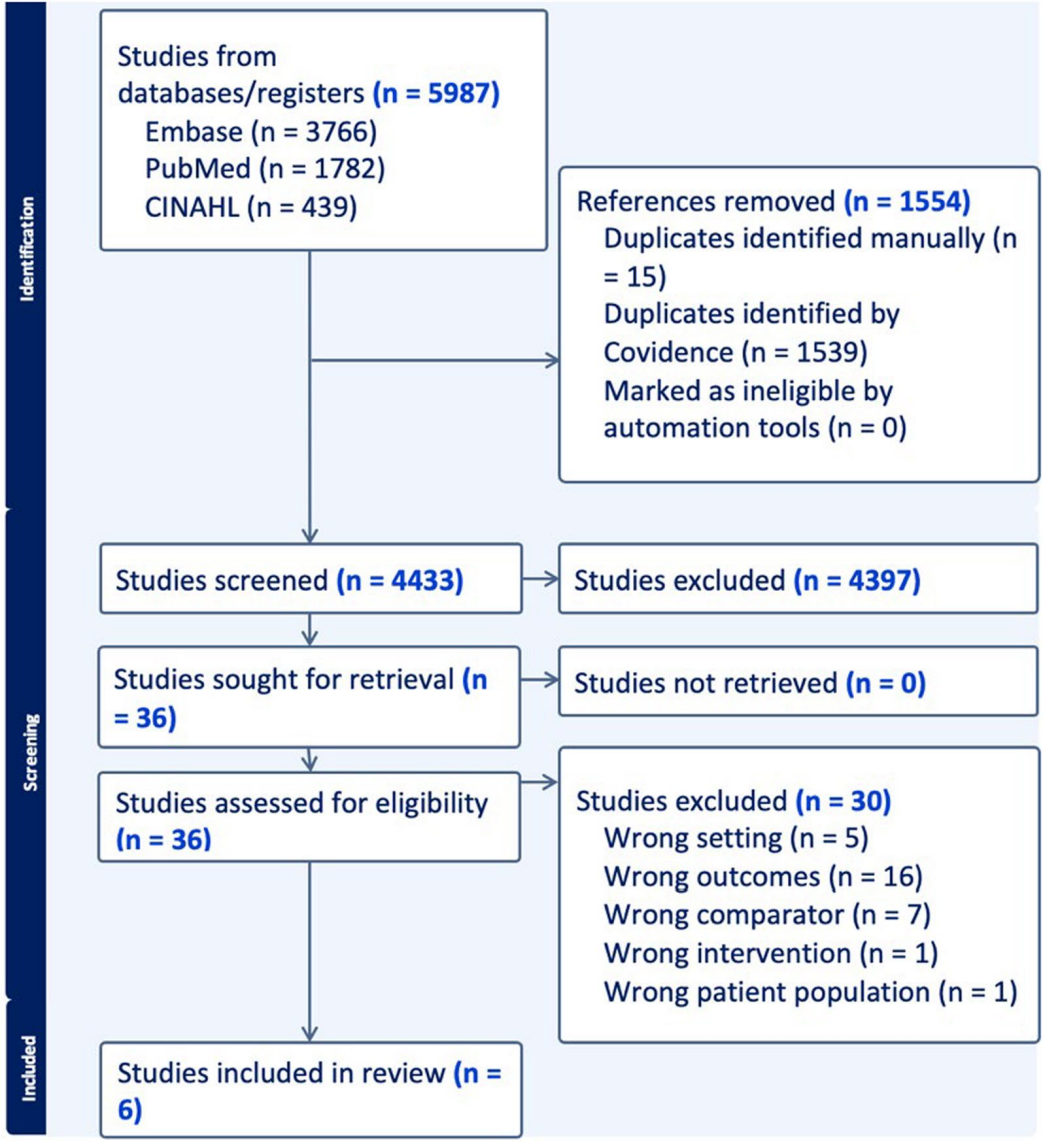
PRISMA flow diagram for literature identification

The search yielded 5,987 citations, and after de-duplication, abstract screening, and full-text review, six studies (< 0.01%) were eligible for analysis (Fig. 1) [19–24]. The overall agreement rate for abstract review was 97%, and Gwet’s AC1 value for inter-rater agreement was 0.97 [17]. There were 144 conflicts (3%) resolved through consensus or third reviewer adjudication.

### Study characteristics and outcome measures

Studies were conducted between 2012 and 2024, across Taiwan, Italy, and the United States (Table 1) [19–24]. Each study was academically sponsored and evaluated a clinical decision support (CDS) tool within an inpatient setting, most commonly at a single center. Most tools (83%) were developed for patients receiving invasive mechanical ventilation (Table 2) [20–24]. Sample sizes ranged between 40 and 2,536 patients. Half of the studies assigned groups by randomization, while the other half evaluated outcomes before and after the implementation of the tool into their health information system (HIS). Three studies (50%) showed statistically significant and clinically meaningful outcome improvements with the AI tool [19, 20, 24]. Outcome measures varied, including duration of mechanical ventilation, intensive care unit (ICU) and hospital length of stay, extubation success, antibiotic duration, changes in treatment, and surrogate decision-making outcomes.

**Table 1:** Study characteristics

First author, year	Country	Funding source	Number of centers	Study setting	Data collection period	Mean age (years ± SD)	Percent female
Gueli et al., 2012	Italy	Academic	1	Geriatric ward	2006–2008	86.0 ± 6.1	NR
Hsu et al., 2013	Taiwan	Academic	1	Respiratory care center	2008–2009	*50.8 ± 31.7	42.6
Cox et al., 2019	United States	Academic	5	Medical and Surgical ICUs	2012–2017	53.4 ± 17.2	36.1
Liao et al., 2022	Taiwan	Academic	1	Respiratory care center	2020–2021	NR	NR
Liu et al., 2022	Taiwan	Academic	1	ICU	2019–2020	66.0 ± 15.8	33.5
Lin et al., 2024	Taiwan	Academic	1	Medical ICU	2019–2023	63.5 ± 16.2	35.8

*SD* standard deviation, *NR* not reported, *ICU* intensive care unit

^*^Author contacted due to discrepancy in values between text and table without response

**Table 2:** Study designs and outcomes

First author	Patient population	Sample size	Randomized	Outcome(s) and effect size of AI tool (mean difference or odds ratio)
Gueli	Lower respiratory tract infection	40	Y	Duration of antibiotic therapy: −8.6 days (95% CI −13.04, −4.16)
				Duration of hospital stay: −8.7 days (95% CI −13.47, −3.93)
				Change in treatment: 0.12 (95% CI 0.03, 0.54)
Hsu	Mechanically ventilated for at least 21 days	312	Y	Duration of MV: −5.2 days (95% CI −7.61, −2.95)
				Successful weaning (no reinitiation of MV within 72 h): 1.46 (95% CI 0.93, 2.29)
Cox	Mechanically ventilated for at least 10 days	277	Y	Physician-surrogate concordance scale: −1.7 (95% CI −8.3, 4.8)
				Decisional conflict scale 0.4 (95% CI 0.0, 0.7)
Liao	Mechanically ventilated for at least 21 days	115	N	Duration of MV among those successfully weaned: −0.5 days (no SD available)
				No reinitiation of MV within 120 h: 1.16 (no SD available)
Liu	(1). Mechanically ventilated on assist control mode for 8–120 h (2). Mechanically ventilated on support mode for 2–13 days	167	N	Duration of MV among those successfully extubated: −0.89 days (95% CI −2.8, 1.02)
				Duration of ICU stay among those successfully extubated: −0.5 days (95% CI −2.78, 1.78)
Lin	(1). Mechanically ventilated on assist control mode for 8–120 h (2). Mechanically ventilated on support mode for 2–13 days	2,536	N	Successful extubation (no reinitiation of MV within 48 h): 1.12 (95% CI 0.93, 1.34)
				Duration of MV: −0.65 days (95% CI −1.32, 0.02)
				Duration of ICU stay: −0.62 days (−1.37, 0.13)
				Duration of hospital stay: −3.88 days (95% CI −5.89, −1.86)

*Y* yes, *N* no, *CI* confidence interval, *MV* mechanical ventilation, *ICU* Intensive Care Unit

### AI tools

**Fig. 2:**
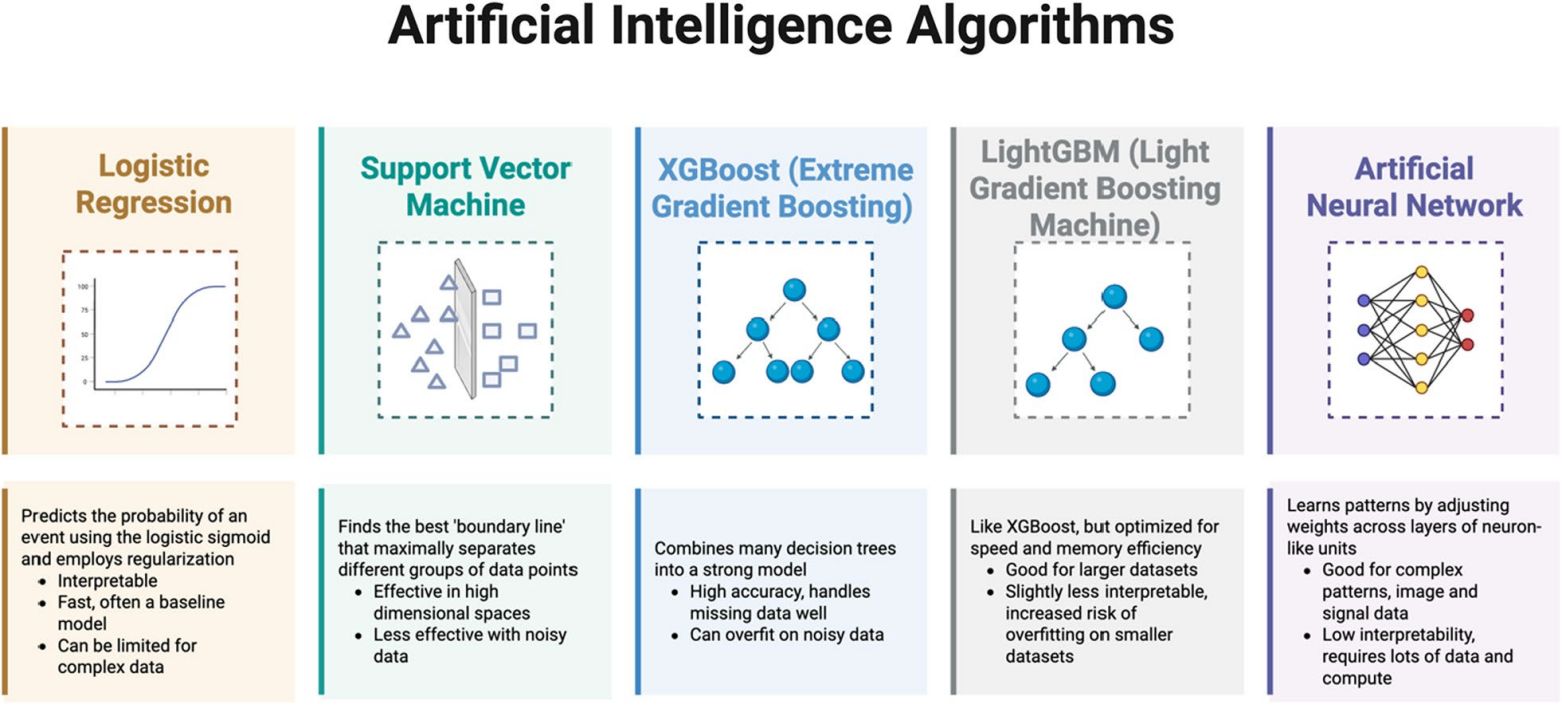
Artificial intelligence algorithms. Artificial intelligence/machine learning models are trained using clinical data to recognize patterns and make predictions. Data are typically split into three sets: a training set to teach the model, a validation set to fine-tune its performance, and a test set to evaluate how well it generalizes. During training, the model minimizes a loss function, a measure of how far off its predictions are, so it can improve over time. Descriptions of the models are not necessarily mutually exclusive

The CDS tools evaluated in the included studies were based on various prediction models, including logistic regression, support vector machines, gradient boosted machines, and artificial neural networks. These algorithms are described in Fig. 2, and the algorithm used for each study is listed in Table 3. The majority of studies (67%) developed tools to predict liberation from invasive mechanical ventilation [20, 22–24]. Data inputs varied, with some tools requiring manual entry to generate predictions, while others automatically integrated data from electronic health records and ventilator systems (Table 3). To support real-time integration with the hospital information system, three studies (50%), from the same center, described a digital infrastructure comprising a graphics processing unit (GPU), database, and web-service servers [22–24]. Their tools refreshed predictions every 60 min and displayed them in time windows tailored to ventilation duration. They also featured interactive interfaces that allowed clinicians to manually adjust inputs, such as suctioning frequency, to visualize predicted outcomes [22–24].

**Table 3:** AI tool design

First author	Data inputs	AI algorithm	AI prediction task	Tool interface	Performance bias assessment?
Gueli	Demographics, comorbidities, symptoms, laboratory values, medications, oxygen	ANN	Resolution index for 19 antibiotics	Graph generated by online neural network modeling software	N
Hsu	RSBI, neurologic scores, infections, duration of MV, tracheotomy, comorbidities, creatinine	SVM	No reinitiation of mechanical ventilation within 120 h	Graphic user interface on clinical decision support software	N
Cox	Age, vasopressors, hemodialysis, platelets, and trauma/non-trauma	Logistic regression	1-year mortality	Interactive web-based decision aid + family meeting	N
Liao	Demographics, comorbidities, severity scores, vital signs, ventilator parameters, neurologic scores, SBT count, suction times, duration of MV	XGBoost	No reinitiation of mechanical ventilation within 120 h	Interactive web-based dashboard integrated with the HIS	N
Liu	Demographics, severity scores, vital signs, ventilator parameters, neurologic scores, SBT count and suction event count	LightGBM	1). Shift from assist control mode to support mode for 48 h 2). No reinitiation of mechanical ventilation within 48 h	Interactive web-based dashboard integrated with the HIS	N
Lin	Demographics, severity scores, vital signs, ventilator parameters, neurologic scores, SBT count and suction event count	LightGBM	1). Shift from assist control mode to support mode for 48 h 2). No reinitiation of mechanical ventilation within 48 h	Interactive web-based dashboard integrated with the HIS	N

*ANN* Artificial Neural Network, *SVM* Support Vector Machine, *XGBoost* Extreme Gradient Boosting, *LightGBM* Light Gradient boosting machine, *RSBI* Rapid Shallow Breathing Index, *MV* mechanical ventilation, *SBT* Spontaneous Breathing Trial, *CDSS* clinical decision support system, *HIS* health information system, *CI* confidence interval, *ICU* Intensive Care Unit, *N* no

### Appraisal of clinical evaluations

**Fig. 3:**
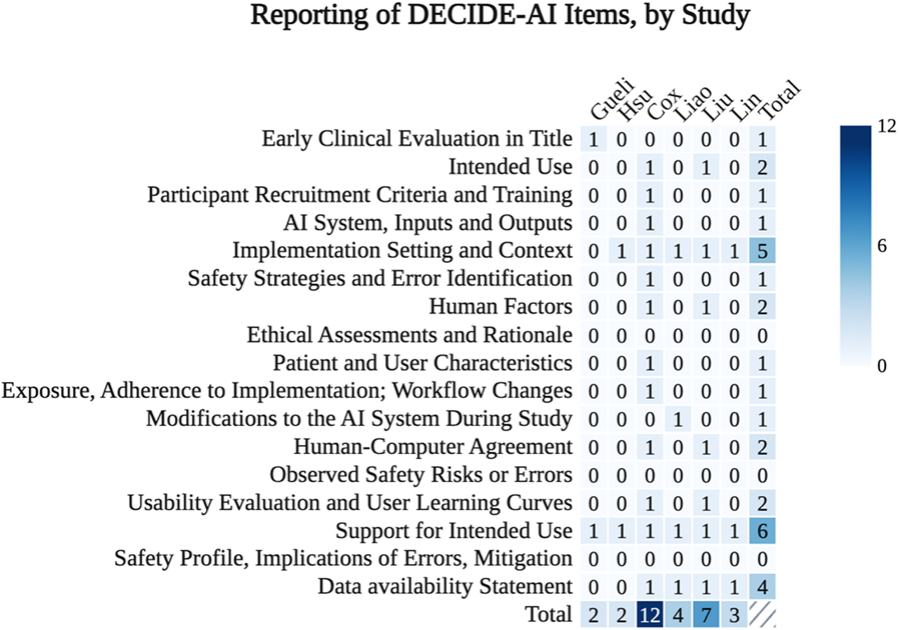
Number of studies reporting AI-Specific Items from DECIDE-AI. Out of 17 Total AI-Specific Reporting Items. Higher numbers represent a more complete evaluation

The included studies reported a median of 3.5 (21%, IQR: 2.25–6.25) of the 17 AI-specific reporting items recommended by the DECIDE-AI guideline (Fig. 3). None of the studies reported whether any errors arose from AI model recommendations, software or hardware malfunctions, or user missteps. Consequently, potential implications for patient care and corresponding mitigation strategies were also not discussed. Additionally, no study described a methodology for evaluating algorithmic fairness, and most studies (67%) did not report how missing data were handled in the development of the AI system.

Only one study (17%) described the characteristics of the users of the support system [21]. Two studies (33%) assessed human–computer agreement and provided or referenced a usability evaluation [21, 23]. For instance, one study noted that the mean session time was 33.4 min, and several secondary outcomes addressed user satisfaction [21]. The other study surveyed physicians post-implementation to assess ease of use, prediction accuracy, and perceived clinical helpfulness. This evaluation, however, did not use a recognized usability framework [23]. Adherence to the intended implementation varied, with one study noting that 91.4% of surrogates completed the tool and the other finding that only one-quarter of ICU physicians reported regularly using the tool.

Four studies disclosed data availability (66%) [21–24]. Almost all studies (83%) described the setting and clinical workflow in which the AI system was implemented. One study also addressed the broader implementation context by referencing national health policy as a factor influencing the definition of the weaning outcome [22]. All studies discussed support for intended use of the AI tool. One study described anticipated advantages of the intervention, which would include potential reductions in cost and hospitalization duration, although implementation-specific costs were not reported [20].

## Discussion

Our review identified six studies from the list of abstracts with qualifying keywords and MeSH terms (< 0.01%) that evaluated AI-driven CDS tools for acute respiratory failure in clinical environments. Notably, four of the six studies focused on weaning from mechanical ventilation. Three studies demonstrated statistically significant outcomes. Most studies were single-center evaluations. Structured performance bias assessments of the underlying AI models were absent. While half of the studies employed randomized designs, none of the studies fully met the DECIDE-AI reporting guideline for early clinical evaluation, and the median number of items reported was 3.5 out of 17 (21%).

### Trends and gaps

Although efforts to evaluate AI tools in clinical practice began more than a decade ago, with the earliest included study published in 2012, we found that only a small number of tools have undergone clinical evaluation since then. Many AI models have been developed with the narrow goal of producing accurate predictions under ideal conditions and have solely evaluated metrics of discrimination and calibration. The studies that have conducted clinical evaluations have offered little guidance for handling user errors or accounting for workup-to-detection burden, number needed to evaluate, system malfunctions, or unexpected or missing clinical inputs. This limited framing has overlooked the complexity and unpredictability of real-world clinical practice. In some cases, the lack of observed clinical benefit may have reflected poor implementation rather than intrinsic model limitations. For example, only one-quarter of ICU physicians reported regularly using one of the AI-driven CDS tools to guide ventilator weaning, while another quarter reported seldom using it. Furthermore, most studies have not reported whether clinicians acted on the tool’s recommendations or what occurred when recommendations were not followed. Without this context, it is difficult to determine whether observed effects, or lack thereof, were due to model performance or its implementation into clinical practice workflows.

#### Usability

A key trend in the included studies was a gap in the evaluation of usability. Usability refers to the extent to which a system enables users to achieve their goals safely, efficiently, and with a positive experience [25]. One useful model of usability frames this through the relationship between three key elements: the user, the task, and the design [26]. It encompasses design principles such as learnability, performance speed, error prevention, and user satisfaction. These features are critical to the adoption and sustained use of AI-driven clinical decision support (CDS) tools in healthcare environments.

Although the studies in this review did not employ structured usability evaluations, the DECIDE-AI framework helped elucidate where key usability principles were incorporated into tool design. For example, the criteria related to system output reporting help elucidate how learnability and performance speed can be supported by consistent graphical user interfaces that display weaning probabilities. Additionally, interactive features that let users manipulate input variables, such as suctioning frequency, to generate prediction changes, illustrate feedback and adaptability. Quantifying exposure to the tool helps identify performance speed, as Cox et al. reported that 91.4% of surrogates completed the tool in a mean session time of 33.4 min. Understanding the timing of the use of the AI tool, such as the real-time dashboard that refreshes predictions every 60 min, also illustrates adaptability, as it updates regularly and displays predictions in time windows tailored to patient-specific mechanical ventilation duration. Usability evaluations, such as physician surveys assessing ease of use, prediction accuracy, and clinical helpfulness, address aspects of learnability, effectiveness, and task conformance. Together, these examples show how usability can be built into AI tools through thoughtful design, but they also highlight the importance of reporting these features.

#### Implementation

Another trend that emerged was the inconsistent reporting of implementation within complex healthcare systems. One structured approach, the Consolidated Framework for Implementation Research (CFIR), emphasizes the multilevel factors that shape implementation, including characteristics of the intervention, the clinical setting, the individuals involved, and the broader processes of addressing barriers and facilitators used to move from low-fidelity prototype prediction models to high-fidelity CDS tools [27].

The DECIDE-AI criteria illuminated elements that map onto CFIR’s core domains. For example, reporting the characteristics of the users who interacted with the AI tool, undertaken by only one study, provides valuable demographic and behavioral context, and addresses the characteristics of individuals domain. Documenting support for intended use, such as describing the potential cost and length of stay improvements from the intervention, addresses part of the intervention characteristics domain. Of note, studies did not report implementation-specific costs, limiting the completeness of this assessment. Additionally, describing the setting where the tool was evaluated, such as the authors who described a robust digital infrastructure, including a graphics processing unit server, database server, and web-service server, used to enable real-time model predictions within the hospital information system, helps describe the available resources and networks and communication needed in the inner setting domain. Describing Taiwanese health policy as a driver for defining the weaning outcome, reflects external incentives and policy alignment that drive the outer setting domain. While these examples suggest early efforts to embed AI tools into practice, the overall reporting of implementation strategies was limited and inconsistent across studies.

### Strengths

This literature search was not limited by publication date, allowing the retrieval of studies dating back to 2006, providing a broader view of how the field has evolved. The search strategy also focused on studies that evaluated AI-driven CDS tools in clinical settings against a comparator, since evaluating readiness for clinical deployment without knowing efficacy offers limited practical insight. Utilizing an established framework, we identified recurring gaps, emerging themes, and design features that may be critical for future success. This framework also allowed us to highlight which tools and studies may hold the most potential for real-world impact going forward. Additionally, these findings are relevant to a range of stakeholders. For clinicians, they underscore the need for AI tools that are useful for clinical workflows. For human factors and implementation science teams, they provide guidance on aligning design with usability and reporting standards. For health system leaders, they emphasize the importance of infrastructure and implementation planning. For patients and families, they demonstrate how well-designed tools may contribute to more informed and collaborative care.

### Limitations

Most of these studies were single-center pilot evaluations; thus, they were not sufficiently powered to observe a clinically significant effect; therefore, outcome effect sizes must be interpreted with caution and validated in larger, appropriately powered clinical studies. In terms of quality assessment, to ensure consistency in evaluation, studies were required to meet all components of a given DECIDE-AI item to receive credit for that category, which may have underestimated partial efforts toward adequate reporting. Additionally, many of the included studies were published before the release of the DECIDE-AI guidelines and did not have the framework available to guide more deliberate design. Most studies prioritized evaluating clinical effectiveness, and the primary objective was not to examine the intervention in depth, limiting the extent of what was reported. Lastly, prediction models from the older studies may not be generalizable today with updates in medical practice.

## Conclusion

This review highlights the limited body of evidence evaluating AI-driven CDS tools for acute respiratory failure in real-world clinical settings. While early use cases such as ventilator weaning show promise, consistent gaps in evaluation and reporting undermine larger clinical deployment. Moving forward, future clinical evaluations should prioritize embedding structured evaluations of safety, usability, human–AI interaction, and implementation strategy. Such an approach is essential to ensure that AI tools meaningfully support clinical decision-making, improve patient outcomes, and ultimately achieve sustained adoption in complex healthcare environments.

## Take home message

Few AI-driven clinical decision support tools for acute respiratory failure have been tested in clinical settings, and those that have, often lack comprehensive quality evaluations. By applying the DECIDE-AI framework, this scoping review identifies key design and reporting gaps that must be addressed to enhance the real-world clinical impact of AI-driven tools.

## Supplementary Information

Supplementary material 1: Table 1. PRISMA Reporting Items for Scoping Reviews. Table 2. Search Terms.

Supplementary material 2: Table 3. Data Extraction Sheet.

## Data Availability

Data extraction form included in supplement. All studies analyzed were published content.

## Abbreviations

AI: Artificial intelligence
ANN: Artificial neural network
ARF: Acute respiratory failure
CDS: Clinical decision support
CDSS: Clinical decision support system
CFIR: Consolidated framework for implementation research
CI: Confidence interval
DECIDE-AI: Developmental and exploratory clinical investigations of decision support systems driven by artificial intelligence
HIS: Health information system
ICU: Intensive care unit
IQR: Interquartile range
ISO: International Organization for Standardization
LightGBM: Light gradient boosting machine
MV: Mechanical ventilation
NCATS: National Center for Advancing Translational Science
NIH: National Institutes of Health
PRISMA-ScR: Preferred reporting items for systematic reviews and meta-analyses extension for scoping reviews
RSBI: Rapid shallow breathing index
SBT: Spontaneous breathing trial
SD: Standard deviation
SVM: Support vector machine
U.S.: United States
XGBoost: Extreme gradient boosting
